# Estimating Vegetation Primary Production in the Heihe River Basin of China with Multi-Source and Multi-Scale Data

**DOI:** 10.1371/journal.pone.0153971

**Published:** 2016-04-18

**Authors:** Tianxiang Cui, Yujie Wang, Rui Sun, Chen Qiao, Wenjie Fan, Guoqing Jiang, Lvyuan Hao, Lei Zhang

**Affiliations:** 1 State Key Laboratory of Remote Sensing Science, Jointly Sponsored by Beijing Normal University and the Institute of Remote Sensing and Digital Earth, CAS, Beijing, China; 2 School of geography and Remote Sensing Sciences, Beijing Normal University, Beijing, China; 3 Beijing Key Lab for Remote Sensing of Environment and Digital Cities, Beijing, China; 4 Northwest Regional Climate Center, Lanzhou, China; 5 School of Atmospheric Sciences, Nanjing University, Nanjing, China; 6 Institute of Remote Sensing and Geographical Information System, Peking University, Beijing, China; Beijing Normal University, CHINA

## Abstract

Estimating gross primary production (GPP) and net primary production (NPP) are significant important in studying carbon cycles. Using models driven by multi-source and multi-scale data is a promising approach to estimate GPP and NPP at regional and global scales. With a focus on data that are openly accessible, this paper presents a GPP and NPP model driven by remotely sensed data and meteorological data with spatial resolutions varying from 30 m to 0.25 degree and temporal resolutions ranging from 3 hours to 1 month, by integrating remote sensing techniques and eco-physiological process theories. Our model is also designed as part of the Multi-source data Synergized Quantitative (MuSyQ) Remote Sensing Production System. In the presented MuSyQ-NPP algorithm, daily GPP for a 10-day period was calculated as a product of incident photosynthetically active radiation (PAR) and its fraction absorbed by vegetation (FPAR) using a light use efficiency (LUE) model. The autotrophic respiration (Ra) was determined using eco-physiological process theories and the daily NPP was obtained as the balance between GPP and Ra. To test its feasibility at regional scales, our model was performed in an arid and semi-arid region of Heihe River Basin, China to generate daily GPP and NPP during the growing season of 2012. The results indicated that both GPP and NPP exhibit clear spatial and temporal patterns in their distribution over Heihe River Basin during the growing season due to the temperature, water and solar influx conditions. After validated against ground-based measurements, MODIS GPP product (MOD17A2H) and results reported in recent literature, we found the MuSyQ-NPP algorithm could yield an RMSE of 2.973 gC m^-2^ d^-1^ and an R of 0.842 when compared with ground-based GPP while an RMSE of 8.010 gC m^-2^ d^-1^ and an R of 0.682 can be achieved for MODIS GPP, the estimated NPP values were also well within the range of previous literature, which proved the reliability of our modelling results. This research suggested that the utilization of multi-source data with various scales would help to the establishment of an appropriate model for calculating GPP and NPP at regional scales with relatively high spatial and temporal resolution.

## Introduction

Gross primary production (GPP) and Net primary production (NPP), which represent the gross carbon fixation and the net carbon input from atmosphere to terrestrial vegetation, play crucial roles in the terrestrial carbon cycle [1,2]. They also serve as key indicators in the evaluation of patterns, dynamics, and processes of terrestrial ecosystem [3]. Therefore, reliable acquisition of GPP and NPP have been considered as indispensable prerequisites for numerous ecological issues. Traditionally, GPP and NPP have been focused on field-based observations of specific species at individual sites, which are usually time consuming and laborious [4,5]. Additionally, it should be noted that these measurements are difficult to extend to large scales due to mismatches between the relatively sparse observations and the spatial heterogeneous [6]. The development of remote sensing has by far improved the ability to study and understand ecosystems with enhanced accuracy [7]. Remote sensing techniques can provide an invaluable opportunity to improve the estimation of GPP and NPP at regional and global scales in a cost effective, efficient and accurate way at multi-spatial and temporal scales [8–11].

Numerous techniques have been developed to estimate GPP and NPP at both regional and global scales during the past several decades: (i) climate productivity models based on relationships between GPP/NPP and climate factors, e.g., Miami model, Thornthwaite Memorial model [12], and Chikugo model [13], etc; (ii) light use efficiency (LUE) models established according to resource balance, e.g., Carnegie, Ames, Stanford Approach (CASA) [14], GLObal Production Efficiency Model (GLO-PEM) [15], and Vegetation Photosynthesis Model (VPM) [16], etc; and (iii) models on the basis of eco-physiological process theories, e.g., CENTURY [17], Terrestrial Ecosystem Model (TEM) [18], and Biome BioGeochemical Cycle (Biome-BGC) model [19], etc. Each group of these models has its advantages and limitations: climate productivity models are well-known for their simplicity while neglect some complex ecological processes as well as the influence of carbon dioxide, soil moisture and nutrient conditions [20]; LUE models take advantage of remotely sensed data, especially when used at regional or global scales, however, these models cannot reflect some critical ecological processes [21]; eco-physiological process models are established based on currently known ecological and biophysical process theories but are restricted by their high complex and large computational demands. Additionally, some input parameters of these process based models are usually difficult to determine [22]. To tackle the limitations mentioned above, models integrated remote sensing techniques with eco-physiological process theories have been developed these years [23,24]. These integrated models occupy the advantages of LUE models and eco-physiological process theories and have been used in both regional and global scales with enhanced reliability and feasibility due to the suitability of remote sensing technology for large scale mapping and the advantage of process models in capturing eco-physiological characteristics [21,25,26].

Typically, there are two ways to combine remote sensing techniques and process models together. One effective approach is to exploit remotely sensed Leaf Area Index (LAI) to combine simplified process models with LUE models, another way is to utilize remotely sensed LAI as an input of a process model. The former is represented by the MODIS GPP/NPP (MOD17A2/A3) algorithm [27] and the latter is represented by the Boreal Ecosystem Productivity Simulator (BEPS) model [1,23]. MODIS GPP/NPP algorithm calculates GPP using a LUE approach with inputs of MODIS LAI and Fraction of Photosynthetically Active Radiation (FPAR) (MOD15A2), land cover categories (MOD12Q1) and meteorological data from NASA’s Global Modeling and Assimilation Office (GMAO). Maintenance and growth respiration components are derived with biomass, annual growth of plant tissues and LAI by incorporating parameters described in the Biome-BGC model, and NPP is obtained by subtracting maintenance and growth respiration items from GPP. BEPS model deploys the theory of FOREST BioGeochemical Cycle (FOREST-BGC) model [28] to quantify biophysical processes and considers remotely sensed LAI and land cover categories as model inputs. Together with meteorological and soil data, BEPS can derive daily or annual NPP.

Although different methods have been performed to generate GPP and NPP at various spatial and temporal scales in former studies [29–31]. We only consider two of the most typically and widely used models, CASA model and MODIS GPP/NPP algorithm, this time. Both of these models have been successfully used in many cases [32,33]. However, it should be noted that CASA model utilizes a biome-independent potential LUE of 0.389 gC MJ^-1^ in estimating global terrestrial NPP [14] while potential LUE varies with biome types actually [15,34]. Additionally, NPP is directly related to the absorbed photosynthetically active radiation (APAR) through LUE in CASA model, which may be inadequate to characterize the respiration items influenced by individual size. As for MODIS GPP/NPP algorithm, it should be noted that daily MODIS Net Photosynthesis (PSNnet) does not include the calculation of growth respiration and maintenance respiration items associated with live wood when compared with NPP [27]. Therefore, this study aims to establish a reliable GPP/NPP algorithm to estimate daily GPP and NPP by integrating remote sensing techniques and eco-physiological process theories, which is designed as part of the Multi-source data Synergized Quantitative (MuSyQ) Remote Sensing Production System. We also consider the availability and practical utility of relatively high resolution data in the establishment of the MuSyQ-NPP algorithm. The objectives of this study are (i) to exploit multi-source and multi-scale data to estimate daily GPP and NPP with relatively high resolution (300 m spatial resolution for a 10-day period); and (ii) to test our model in Heihe River Basin, China and to derive growing season GPP and NPP of 2012. The findings of this study serve to the establishment of an appropriate model for calculating GPP and NPP at regional and global scales with relatively high spatial and temporal resolution and a better understanding of GPP and NPP in an arid area of Heihe River Basin.

## Study Area and Materials

### Overview of the Study area

**Fig 1:**
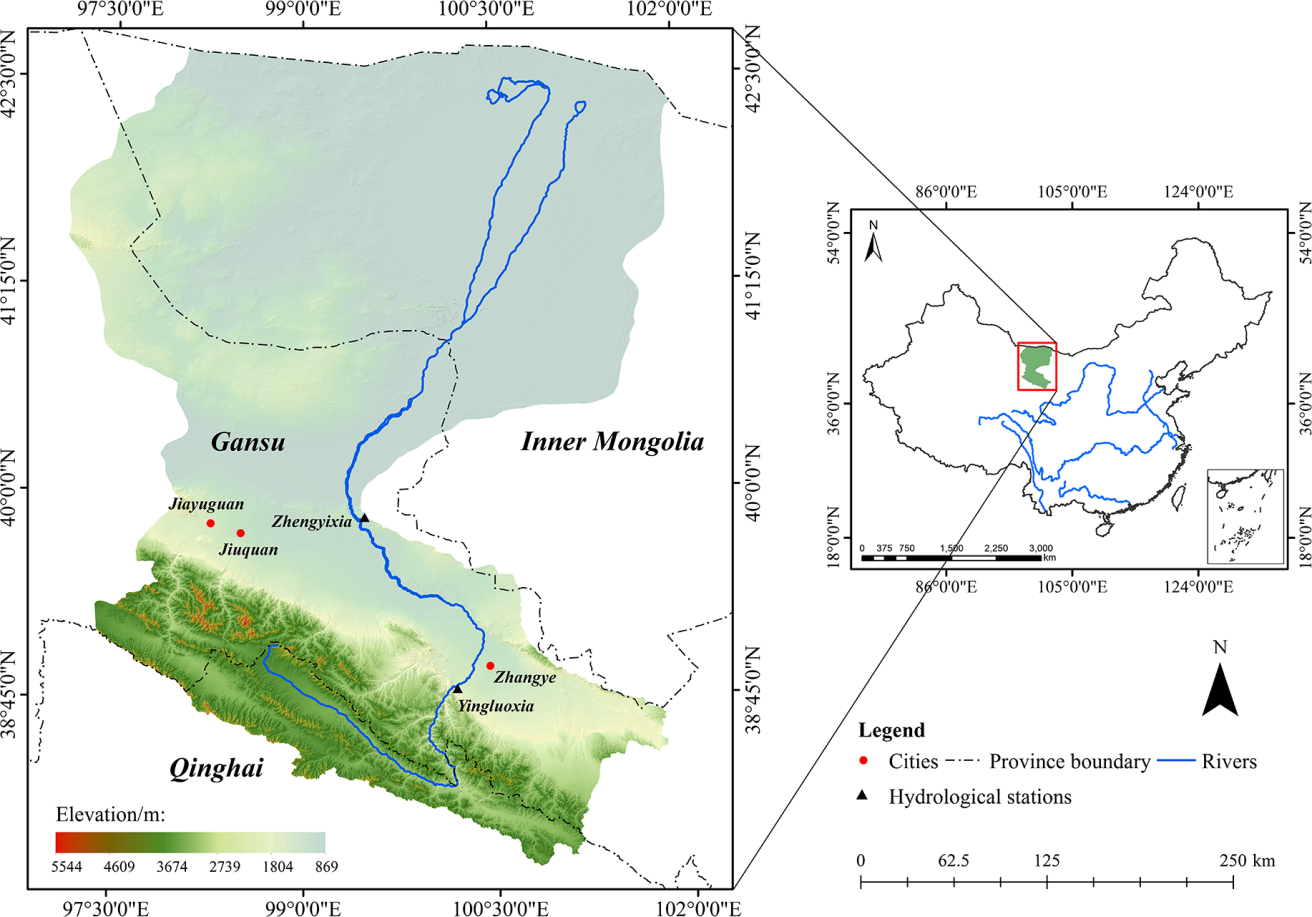
Location and topography of the Heihe River Basin, China.

Heihe River Basin is located in a typical arid and semi-arid region of northwest of China (37°41′ N~42°42′ N and 96°42′ E~102°00′ E) (Fig 1). The total basin area is approximately 128900 km^2^ with a mainstream length of 821 km [35]. Topography of this area varies significantly from south to north with an average altitude over 1200 m [36]. Generally, three sections with different natural, ecological and climate characteristics were divided in Heihe River Basin, including the upstream, midstream and downstream [37]. Among which, the upstream is characterized by the mountainous terrains from Qilian Mountains to Yingluoxia Canyon covered by alpine meadow, the midstream, from Yingluoxia Canyon to Zhengyixia Canyon, is characterized by oases with irrigated agriculture, and the downstream is mainly covered by Gobi desert with reduced runoffs [38]. According to previous published literature, average annual air temperature is -3.1–3.6°C in the upstream region, 7–8.2°C in the midstream region, and 8–10°C in the downstream region, average annual precipitation is 350–450 mm, 80–120 mm and 40–60 mm in the upstream, midstream and downstream regions, respectively [39].

### Data and data processing

#### Remotely sensed data

Remotely sensed FPAR and LAI data derived from HJ-1/CCD images by Fan et al. [40] and Liao et al. [41], with a spatial resolution of 30 m and a temporal resolution of 1 month, covering May to October, 2012, were used in this study. These two remotely sensed products were considered of good quality after evaluated with field measurements [40,41]. Land cover categories data with a resolution of 30 m, acquired with HJ-1/CCD images [42] were also used in our research. The land cover categories data was also considered reliable after manually checked by using very high spatial resolution remotely sensed data and time series HJ-1/CCD data [42]. All these three types of data came from the Heihe Watershed Allied Telemetry Experimental Research (HiWATER) (http://westdc.westgis.ac.cn/data) and were openly accessible.

To generate GPP and NPP at 300 m spatial resolution, remotely sensed FPAR, LAI and land cover categories data with 30 m spatial resolution were all spatially aggregated to 300 m resolution. It should be noted that different aggregating strategies were performed to these three types of data: For FPAR and LAI data, the averaged value in each 300 m × 300 m area was used in the aggregating approach, and for land cover categories, we applied an aggregating method based on maximum area rule.

#### DEM data

DEM data used in this study came from the United States Geological Survey (USGS) HYDRO1k data sets (https://lta.cr.usgs.gov/HYDRO1K). HYDRO1k is developed at the USGS Earth Resources Observation and Science (EROS) Center. It provides hydrologically correct DEM along with ancillary data sets at a resolution of 1 km. In this study, we used the 1 km DEM to generate 300 m DEM using bilinear interpolation algorithm.

#### Meteorological data

Meteorological data were derived from Global Land Data Assimilation System (GLDAS) products (http://ldas.gsfc.nasa.gov/gldas/GLDASgoals.php). GLDAS generates land surface states and fluxes data using advanced land surface model and data assimilation techniques with both satellite and ground based observational data [43]. It provides a set of 1.0 degree and a set of 0.25 degree products, covering 1979 to present and 2000 to present, respectively. Temporal resolution for GLDAS products is 3-hourly. Monthly products are also available by temporal averaging of the 3-hourly products. We used five GLDAS-provided parameters in our study, including the 0.25 degree 3-hourly near surface air temperature, near surface specific humidity, surface incident shortwave radiation, net shortwave radiation, and net longwave radiation.

GLDAS data covering the growing season between April 30th and October 26th, 2012 (Day of the Year: DOY 121 and 300, respectively) were used in this study. The 10-day averaged daily radiation items (surface incident shortwave radiation, net shortwave radiation, and net longwave radiation) (W m^-2^) were obtained by calculating their mean values in a 10-day period. Daily surface incident shortwave radiation item (J m^-2^) were obtained by multiplying the 10-day averaged daily surface incident shortwave radiation (W m^-2^) by 24 and 3600. Daily near surface air temperature and daily near surface specific humidity items, were generated by averaging the 3-hourly GLDAS data in a 10-day period.

All daily items were converted from 0.25 degree to 300 m using bilinear interpolation algorithm. It should be pointed out that we also considered the impact of altitude in the processing of temperature: sea level air temperate with 0.25 degree spatial resolution was firstly generated using near surface air temperature and DEM data of 0.25 degree under the assumption that temperature lapse rate is 6.5°C km^-1^ [44]. The 300 m near surface air temperature was then obtained using interpolated sea level air temperature and DEM of 300 m.

Daily photosynthetically active radiation (PAR) for a 10-day period used in this study were calculated by multiplying daily surface incident shortwave radiation by 0.5 [45].

#### Forest biomass

Forest biomass with 1 km spatial resolution came from the forest carbon density distribution data of 1998–2003, which was acquired from the Chinese Data Sharing Infrastructure of Earth System Science (http://www.geodata.cn) [46].

Bilinear interpolation algorithm was performed to convert forest biomass data from 1 km to 300 m. During this procedure, invalid values were replaced by biome-specific averaged biomass values generated from the forest carbon density distribution data by referring to the land cover categories data.

#### Ground-based field data

**Fig 2:**
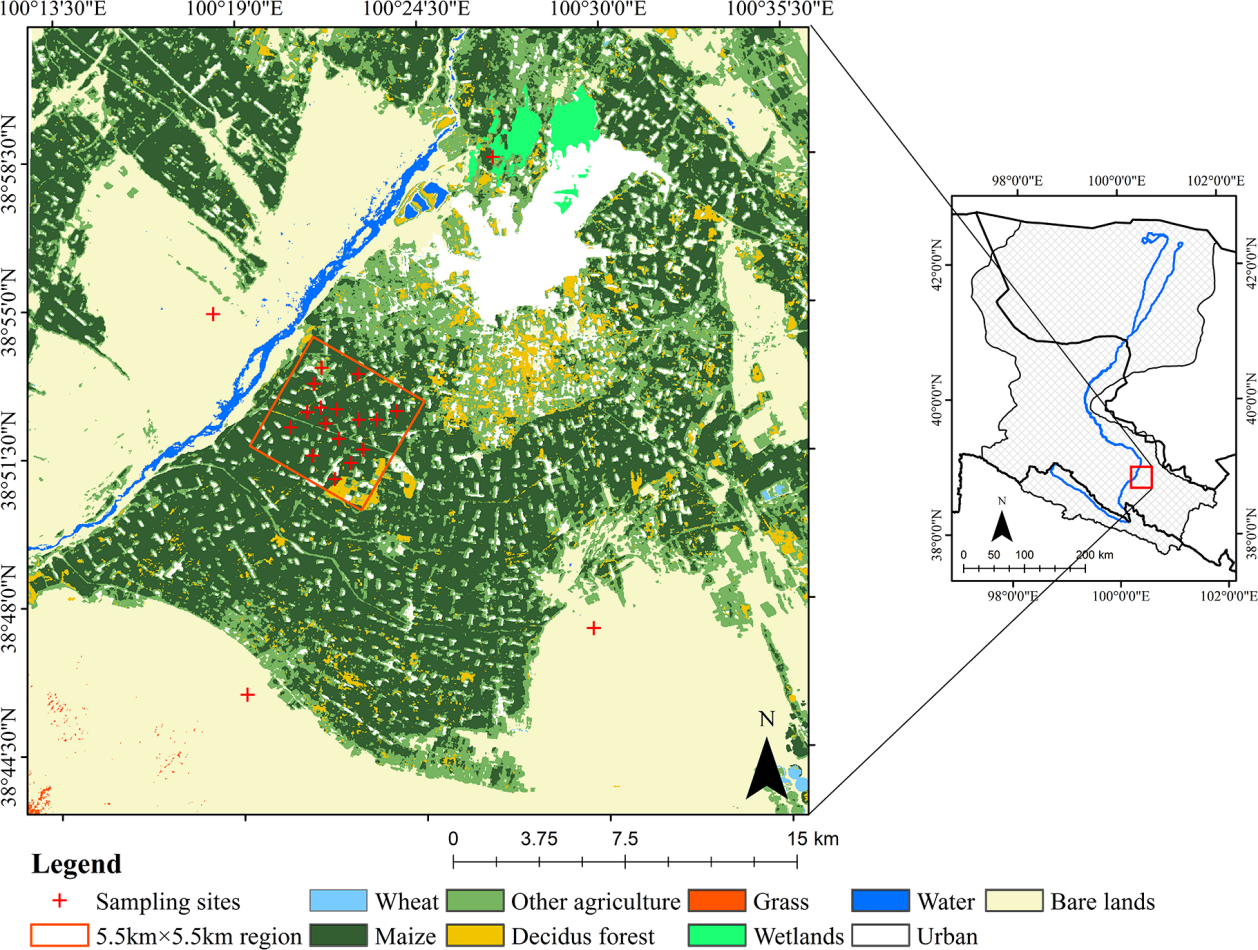
Spatial distribution of field sampling sites.

Ground-based field data used in this study were derived from the MUlti-Scale Observation EXperiment on Evapotranspiration over heterogeneous land surfaces 2012 of the Heihe Watershed Allied Telemetry Experiment Research (HiWATER-MUSOEXE). HiWATER-MUSOEXE encompasses two nested matrices in the midstream area of Heihe River Basin: a core experimental area consists seventeen sampling plots distributed according to landscape situations, agricultural structures and irrigation status in a 5.5 km × 5.5 km region, and a large experimental area consists one superstation and four ordinary sites in a 30 km × 30 km region [47,48,49]. In our study, twenty field observation sites, including fourteen croplands (mainly maize), one orchard, one vegetable field, one desert, one desert steppe, one Gobi, and one wetland, were used to evaluate the retrieved GPP. All the observation sites were distributed in the midstream region of Heihe River Basin (Fig 2). Half-hourly data of CO_2_ flux and associated meteorological variables were obtained. Gap-filling and flux-partitioning were processed using the Edire software package (http://www.geos.ed.ac.uk/abs/research/micromet/EdiRe). By considering data availability, GPP between June 9th and September 16th, 2012 (DOY 161 and 260, respectively) were obtained by partitioning the observed net flux into GPP and ecosystem respiration according to Coops et al. [50], Wang et al. [51], and Zhang et al. [52].

No specific permissions were required in the field measurements in this study. The sampling sites did not include endangered or protected species.

## Methodology

### GPP estimation

Due to its efficiency in characterizing consistent ecosystem processes across various vegetation types, LUE model was used to derive GPP in the MuSyQ NPP algorithm. LUE model is built upon two fundamental assumptions: one is that GPP is directly related to APAR through LUE, where LUE is defined as the amount of carbon produced per unit of APAR; the other is that actual LUE may be reduced below its potential value by environmental stresses such as low temperatures or water shortages [53]. Thus, daily GPP can be described as:

GPP=APAR×ε (1)

APAR=FPAR×PAR (2)

where *APAR* is the absorbed photosynthetically active radiation (MJ m^-2^) per day, which can be obtained from daily *PAR* (MJ m^-2^) and *FPAR*, *ε* is the LUE (gC MJ^-1^).

To calculate daily GPP for a 10-day period, model inputs were all converted to a 10-day averaged daily scale with 300 m spatial resolution using methods described above. As LAI and FPAR data used in this study were obtained at monthly scale, we assumed these two parameters remain constant within each month. Therefore, monthly LAI and FPAR data were used to represent daily conditions for each month this time.

#### Estimation of LUE

As actual LUE may be reduced below its potential value by environmental stresses, the quantification of LUE can be described as:

$$\varepsilon =\varepsilon_{\text{max}}\times f_{1}(T)\times f_{2}(\beta )$$ (3)

where *ε*_max_ is the biome-specific potential LUE (gC MJ^-1^), *f*_1_(*T*) and *f*_2_(*β*) represents the down-regulation effects of temperature and water conditions on *ε*_max_, respectively.

To quantify LUE, we firstly established a biome-specific potential *ε*_max_ Look-Up Table (LUT) by referring to land cover categories according to the Biome Properties Look-Up Table (BPLUT) of MODIS GPP/NPP algorithm [27]. Since MODIS GPP/NPP algorithm deploys a unique potential *ε*_max_ value for crops without distinguishing C3 and C4 species, we used a potential *ε*_max_ value of 1.30 gC MJ^-1^ for C3 crops and a potential *ε*_max_ value of 1.70 gC MJ^-1^ for C4 crops, respectively, in the MuSyQ-NPP algorithm [54]. The temperature- and water-limited effect on *ε*_max_ were generated according to the theory of CASA model [14].

As described in CASA model, effects of temperature stress *f*_1_(*T*) can be divided into two terms, *T*_ε1_ and *T*_ε2_:

$$f_{1}(T)=T_{\varepsilon 1}\times T_{\varepsilon 2}$$ (4)

where *T*_ε1_ represents the depress of *ε* when temperature is above or below the optimum temperature, and *T*_ε2_ reflects the phenomenon that the efficiency of light utilization would be depressed when plants growing at temperatures displace from their optimum temperature [14]. These two items are defined as follows:

$$T_{\varepsilon 1}=0.8+0.02T_{\text{opt}}-0.0005T_{\text{opt}}{}^{2}$$ (5)

$$T_{\varepsilon 2}=1.1814/\left\{\left[1+e^{0.2(T_{\text{opt}}-10-T)}\right]\left[1+e^{0.3(-T_{\text{opt}}-10+T)}\right]\right\}$$ (6)

where *T*_opt_ represents the optimum temperature, which is defined as the mean air temperature in the month when LAI reaches its maximum for the year. For our study area, LAI was found reaching its maximum at August after conducting a seasonal comparison among MODIS LAI products. Thus, the mean air temperature of August was considered as *T*_opt_. For Heihe River Basin, *T*_opt_ ranges from 21.8 to 26.7°C.

The limited effect of water conditions on plant photosynthesis *f*_2_(*β*), ranging between 0.5 and 1, is derived following the algorithm:

$$f_{2}(\beta )=0.5+0.5E/E_{\text{p}}$$ (7)

where *E* and *E*_p_ represents actual and potential evapotranspiration, respectively. Consequently, accurate estimates of GPP will depend strongly on the accuracy of the estimated evapotranspiration.

Former studies suggested that Penman-Monteith (P-M) equation is a biophysically sound and robust framework for estimating daily evapotranspiration at regional and global scales using remotely sensed data [55,56]. In the MuSyQ-NPP algorithm, we used a modified P-M approach with biome-specific canopy conductance to estimate daily actual evapotranspiration [57,58]. Evapotranspiration can be partitioned into soil evaporation and canopy transpiration by partitioning available energy *A* using vegetation cover fraction *F*_c_. Available energy components for canopy (*A*_canopy_) and soil (*A*_soil_) surface were generated using:

$$A_{\text{canopy}}=F_{\text{c}}\times A$$ (8)

$$A_{\text{soil}}=(1-F_{\text{c}})\times A$$ (9)

where *A* is approximated as net radiation *R*_n_ consisting both net shortwave radiation and net longwave radiation as soil heat flux is nearly zero in daily scale. To reduce numbers of inputs and to simplify the procedure and algorithm, we used FPAR as a surrogate of *F*_c_ in our model to allocate *A* to the canopy and soil by referring to Los et al. [59] and Mu et al. [60].

The P-M equation is used to generate vegetation transpiration as:

$$\lambda E_{\text{canopy}}=\frac{\Delta A_{\text{canopy}}+\rho C_{\text{p}}VPDg_{\text{a}}}{\Delta +\gamma (1+g_{\text{a}}/g_{\text{c}})}$$ (10)

where *λE*_canopy_ (W m^-2^) is the latent heat flux of canopy, △ = d(*e*_sat_)/d*T* (Pa K^-1^) is the slope of the curve relating saturated water vapor pressure *e*_sat_ (Pa) to air temperature *T* (K), *ρ* (kg m^-3^) is air density, *C*_p_ (J kg^-1^ K^-1^) is the specific heat of air at constant pressure, *VPD* = *e*_sat_—*e* (Pa) is the vapor pressure deficit of air, *g*_a_ (m s^-1^) is aerodynamic conductance, *γ* (Pa k^-1^) is psychometric constant, and *g*_c_ (m s^-1^) is canopy conductance.

As P-M equation is not sensitive to the variation of aerodynamic conductance when it is in the range of 0.0010–0.033 m s^-1^, the aerodynamic conductance *g*_a_ was assigned constant values of 0.033, 0.0125, 0.010 and 0.010 m s^-1^ for forests, shrubs, grassland and croplands, respectively in our model by referring to Zhang et al. [61]. The canopy conductance, *g*_c,_ is influenced by *g*_sx_ (maximum stomatal conductance of leaves at the top of canopy), LAI, absorbed shortwave radiation, and *VPD*, which can be described as:

$$g_{\text{c}}=\frac{g_{\text{sx}}}{K_{\text{Q}}}\text{ln}\left[\frac{Q_{\text{h}}+Q_{50}}{Q_{\text{h}}\text{exp}(-K_{\text{Q}}\text{LAI})+Q_{50}}\right]\left[\frac{1}{1+VPD/D_{50}}\right]$$ (11)

where *K*_Q_ is the extinction coefficient for photosynthetically active radiation, *Q*_h_ is the photosynthetically active radiation at the top of canopy, *Q*_50_ and *D*_50_ are the values of absorbed photosynthetically active radiation and water vapor deficit when stomatal conductance is half its maximum value, respectively. Values of *g*_sx_ was considered as biome specific and assigned to 0.0047, 0.0028, 0.0032 and 0.0032 m s^-1^ for forests, shrubs, grassland and crops, respectively. *K*_Q_, *Q*_50_, and *D*_50_ were assigned to 0.6, 2.6 MJ m^-2^ d^-1^ and 800 Pa, respectively [61].

Soil evaporation is calculated using a soil evaporation equation described in Zhang et al. [58] and Mu et al. [60], which is described as:

$$\lambda E_{\text{soil}}=RH^{\left(VPD/k\right)}\frac{\Delta A_{\text{soil}}+\rho C_{\text{p}}VPDg_{\text{a}}}{\Delta +\gamma \times g_{\text{a}}/g_{\text{totc}}}$$ (12)

where *λE*_soil_ (W m^-2^) is the latent heat flux of soil, *RH* is the relative humidity of air with values ranging from 0 to 1, *k* (Pa) is a parameter to fit the complementary relationship and is empirically adjusted for different vegetation types, and *g*_totc_ (m s^-1^) is the corrected value of total aerodynamic conductance as described by Zhang et al. [56].

Potential evapotranspiration, *E*_p_, is calculated using the Priestley and Taylor (P-T) equation [62] by considering data availability:

$$\lambda E_{\text{p}}=\phi A\frac{\Delta }{\Delta +\gamma }$$ (13)

where the P-T coefficient *φ* was set to 1.26 following Priestley and Taylor [62].

### Autotrophic respiration estimation

Autotrophic respiration (*R*_a_) can be separated into maintenance respiration (*R*_m_) and growth respiration (*R*_g_), the former represents the energy required to maintain biomass, and the latter represents the energy that converting assimilates into new structural plant constituents:

$$R_{\text{a}}=R_{\text{m}}+R_{\text{g}}=\sum_{i}\left(R_{\text{m},i}+R_{\text{g},i}\right)$$ (14)

where *i* represents a plant component, *i* = 1, 2, 3, namely, leaves, stems, and roots, respectively. Maintenance respiration is temperature-dependent:

$$R_{\text{m},i}=M_{i}r_{\text{m},i}Q_{10,i}{}^{(T-T_{\text{b}})/10}$$ (15)

where *M*_*i*_ (kgC m^-2^) is the biomass of plant component *i*, *r*_*m*,*i*_ (kgC kg^-1^) is maintenance respiration coefficient for component *i*, *Q*_10,*i*_ is the temperature sensitivity factor, *T* (°C) is the daily average temperature and *T*_*b*_ (°C) is the base temperature. For the presented MuSyQ-NPP algorithm, values of maintenance respiration coefficient *r*_*m*,*i*_, and temperature sensitivity factor *Q*_10,*i*_ were treated as biome-dependent and determined by referring to the BPLUT of MODIS GPP/NPP algorithm [27].

To model maintenance respiration, non-forest and forest lands are treated separately. Maintenance respiration for non-forest lands is generated with LAI and specific leaf area (SLA) using:

$$R_{\text{m}}=\text{LAI}/\text{SLA}\times 0.5\times 2^{(T-T_{\text{b}})/10}$$ (16)

where SLA was obtained from the BPLUT of MODIS GPP/NPP algorithm [27] in our model.

Maintenance respiration for forest lands are obtained by calculating maintenance respiration of leaf, stem, and root, separately. All of these three items depend strongly on leaf, stem, and root mass, respectively. Among which, leaf mass is estimated from LAI and SLA as:

$$M_{1}=\text{LAI}/\text{SLA}$$ (17)

Stem and root mass are generated using:

$$M_{2}=biomass/(1+y)$$ (18)

$$M_{3}=y\times M_{2}$$ (19)

where *y*, an ecophysiological biome-specific constant, is obtained from the BEPS model [23], represents the ratio of root to stem mass.

The leaf, stem, fine root and coarse root respiration items were calculated separately by referring to the BEPS model [23] in our model.

Growth respiration is considered to be independent to temperature and is proportional to GPP:

$$R_{\text{g}}=\gamma (GPP-R_{\text{m}})$$ (20)

where *γ* is the growth respiration coefficient and defined as 0.25.

### NPP estimation

NPP represents the net flow of carbon from the atmosphere to plants, and defines as a balance between GPP and autotrophic respiration. Thus, NPP can be expressed as:

$$NPP=GPP\;-\;R_{\text{a}}$$ (21)

### Model Evaluation

To assess the performance of MuSyQ-NPP algorithm, three approaches were applied: to compare directly with the ground-based GPP, to compare with the MODIS GPP product (MOD17A2H) and to compare with NPP values reported in recent literature for the same study area or similar biomes. The statistical indexes of correlation coefficient (R) and overall Root Mean Square Error (RMSE) were used to quantify the modelling GPP results.

## Results

### Modelling GPP and NPP

**Fig 3:**
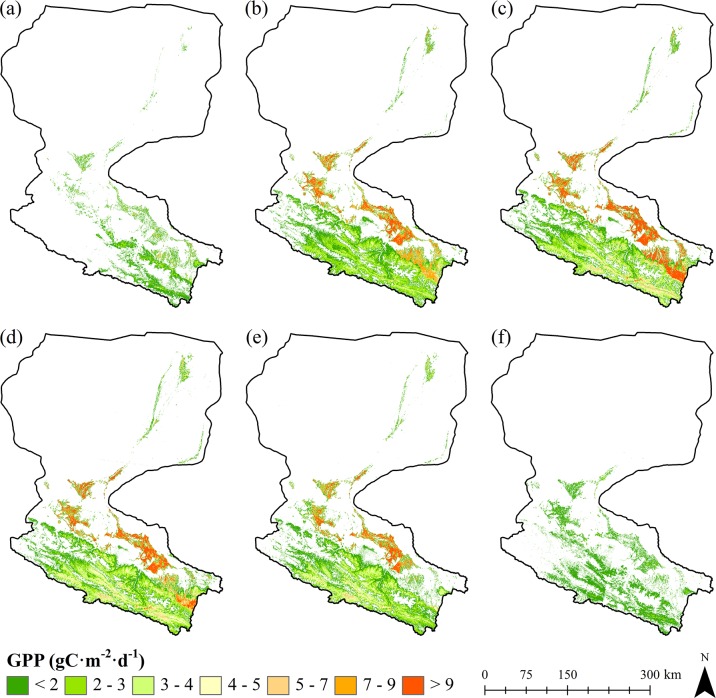
Daily GPP for a 10-day period of Heihe River Basin. (a) DOY 131–140. (b) DOY 161–170. (c) DOY 191–200. (d) DOY 221–230. (e) DOY 251–260. (f) DOY 281–290.

**Fig 4:**
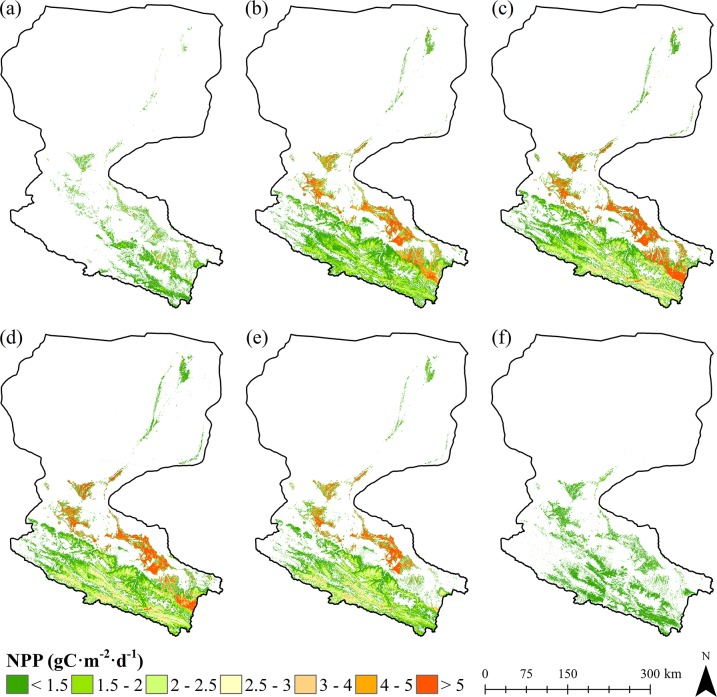
Daily NPP for a 10-day period of Heihe River Basin. (a) DOY 131–140. (b) DOY 161–170. (c) DOY 191–200. (d) DOY 221–230. (e) DOY 251–260. (f) DOY 281–290.

The maps of growing season GPP and NPP reveal clear spatial and temporal patterns in their distribution over Heihe River Basin. As shown in Fig 3 and Fig 4, both GPP and NPP are relatively high in the southern part of mountainous areas in the upstream, oasis areas in the midstream, and riparian areas adjacent to the water body in the downstream. Among which, the oasis areas in the midstream covered with crops occupy the highest values of GPP and NPP, followed by the mountainous areas in the upstream covered with grasses. The downstream regions covered with deserts and Gobi demonstrate the lowest production values. Temporal dynamic patterns of GPP and NPP reveal both of these datasets increase over time initially and decreases after reaching their maximum on July and August. The spatial and temporal patterns of GPP and NPP corresponds to the vegetation growth characteristics in Heihe River Basin.

**Fig 5:**
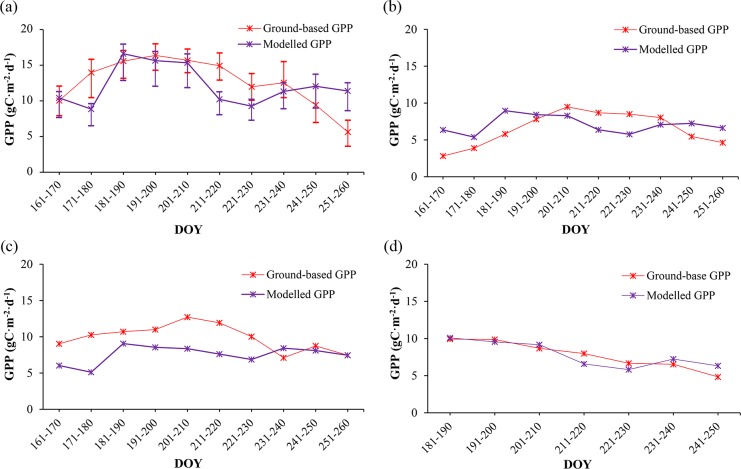
Temporal dynamic patterns of modelled and ground based GPP during the growing season of 2012. (a) croplands. (b)orchard. (c)vegetable field. (d)wetland.

**Fig 6:**
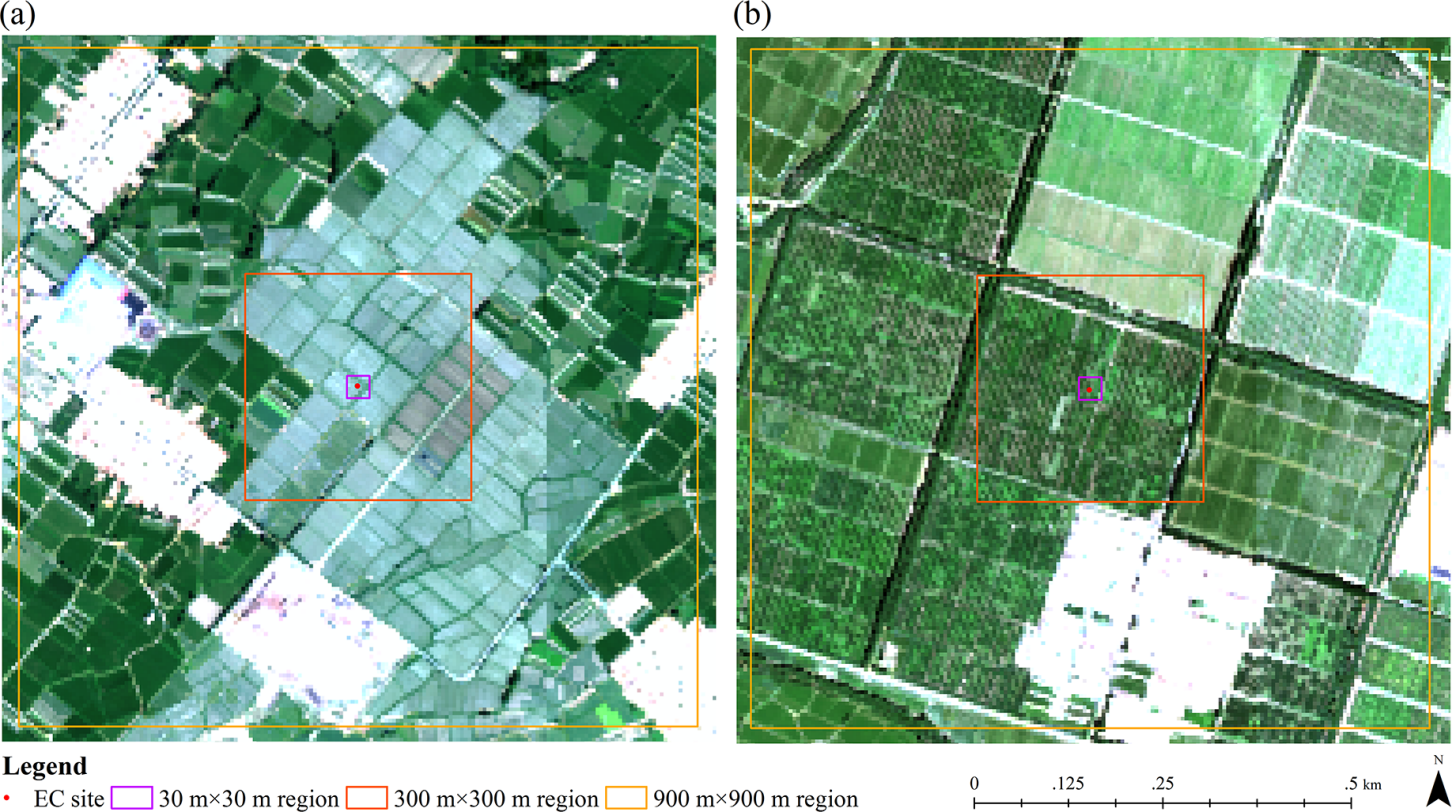
Land surface conditions around the EC sites. (a) orchard. (b) vegetable field.

Among the twenty field measurement sites, we chose seventeen sites covered mainly by vegetation to analyze the temporal dynamic patterns of GPP during the growing season of 2012, which included fourteen croplands, one orchard, one vegetable field and one wetland. Both modelled and observed GPP were obtained. It should be pointed out that we used the mean values in a 3 pixels × 3 pixels window around each sampling site as modelled results to decrease the co-registration errors between image and sampling sites in this study. As shown in Fig 5, both modelled and observed GPP for croplands, orchard and vegetable field increase initially and then decrease after reaching their maximum around July (DOY 181–201). During this period, mountain snow in the southern part of our study region melt as temperature increases, which creates a better water supply. Additionally, the increased temperature, precipitation and solar influx in summer can also lead to higher GPP and NPP values. After August, both temperature and precipitation decrease over time. As environmental conditions getting worse for vegetation growing, GPP and NPP decreases subsequently. Fig 5 also indicates that the modelled GPP have more dramatically increase and decrease patterns than the observed ones, such as the significant differences between DOY 171–180 and DOY 181–190, DOY 201–210 and DOY 211–220, DOY 231–240 and DOY 241–250, which may be attributed to the monthly scaled LAI and FPAR data used in our model. Besides the mismatch between the monthly inputs and daily outputs, the significant differences between the modelled and observed GPP for orchard and vegetable field can also be attributed to their spatial distributions. Compared with croplands and wetland, both these two land cover categories occupy relatively small areas around the EC sites in our study region. Although ground surfaces at the EC sites and around them are relatively homogeneous, land cover categories demonstrate significant variability within large areas like 900 m × 900 m (3 pixels × 3 pixels) especially for orchard and vegetable field with small areas (Fig 6). Fig 6 illustrates the Compact Airborne Spectrographic Imager (CASI) image derived from the HiWATER-MUSOEXE with 5 m spatial resolution acquired on June 29th, 2012 [63], together with the 30 m × 30 m, 300 m × 300 m, and 900 m × 900 m boundaries around the EC sites of orchard and vegetable field. As shown in Fig 6, croplands and urban areas occupy significant proportions within the 900 m × 900 m regions around both the orchard and vegetable field sites. The significant variability of land cover categories will lead to some uncertainties and result in significant differences between the modelled and observed results.

**Fig 7:**
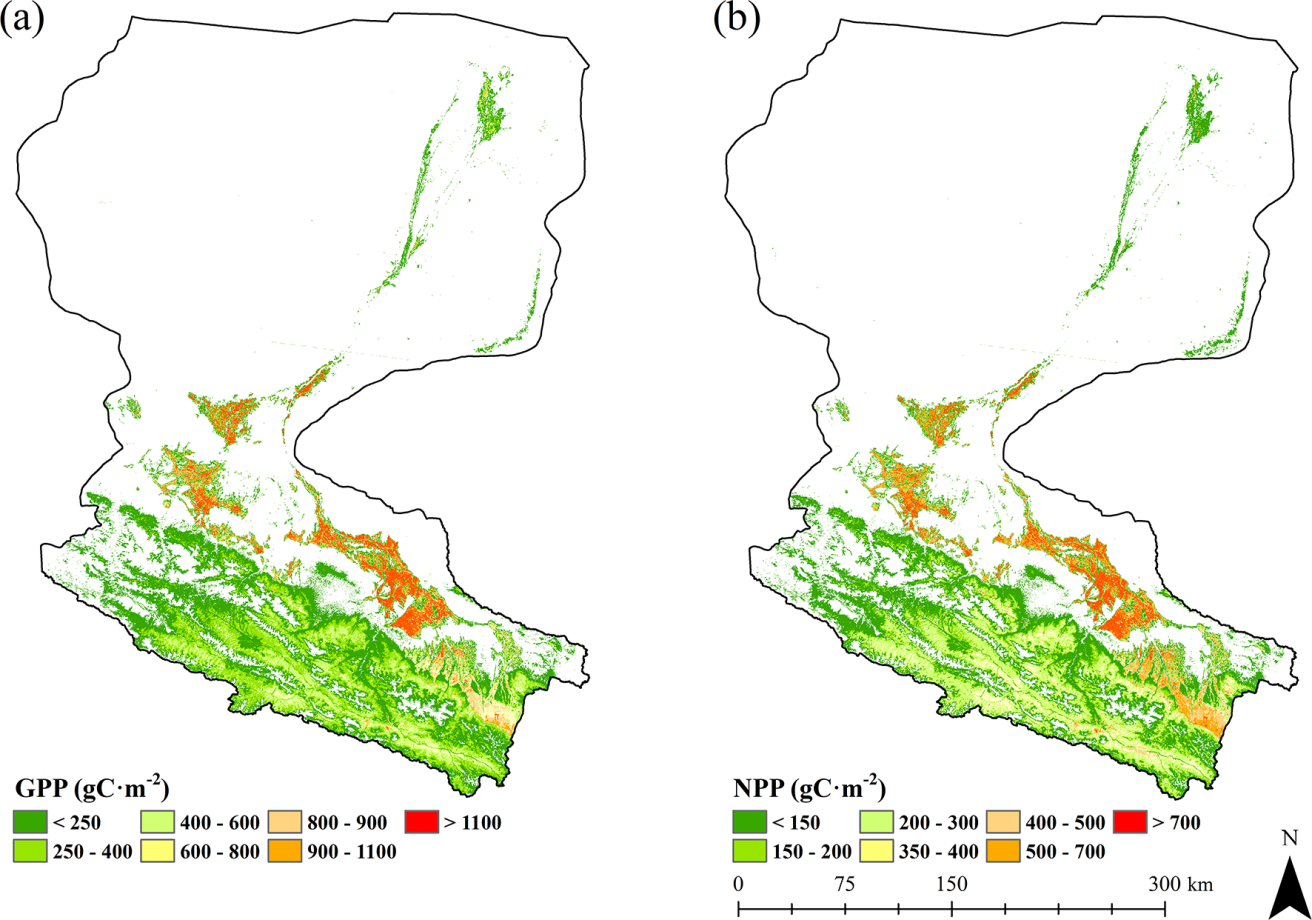
Spatial distribution of GPP and NPP of Heihe River Basin during the growing season of 2012. (a) GPP. (b) NPP.

We also generated GPP and NPP over the whole growing season. As shows in Fig 7, the growing season GPP and NPP are significantly high in the areas covered by croplands (mainly maize). Due to the fact that maize (C4 crop) occupies a significantly higher potential LUE [33,54], we consider the derived higher GPP and NPP over croplands are partly attributed to the higher potential LUE used in the model. In addition, although precipitation is the major limiting factor for vegetation growth in arid areas, croplands are influenced by irrigation during the growing period. Thus, compared with other land cover categories, croplands occupy a better water supply, water stress of these areas would be much smaller and conditions for vegetation growth would be much better, which will definitely lead to higher GPP and NPP values.

In general, there is a distinct gradient in the distribution of GPP and NPP along the upstream, midstream, and downstream of Heihe River Basin from south to north during the growing period. The upstream regions covered by sparse grasses show relatively low production with values of 57–543 gC m^-2^ for GPP and 31–271 gC m^-2^ for NPP, the midstream areas of croplands demonstrate much higher values of GPP and NPP, 733–1433 gC m^-2^ and 443–847 gC m^-2^, respectively, and the downstream regions covered with shrubs adjacent to the water body show the lowest production values of 47–317 gC m^-2^ for GPP and 23–137 gC m^-2^ for NPP. This pattern is driven primarily by both temperature and water conditions presented in Heihe River Basin: The upstream region is characterized by mountainous terrains with average annual temperature of -3.1 to 3.6°C [39]. Although this area occupies relatively large amount of precipitations (350–450 mm annually), relatively low temperature of these areas inhibits the growth of vegetation. In midstream regions, average annual temperature is 7 to 8.2°C [39], which is significantly higher than that of the upstream region. Additionally, irrigations for artificial fields during growing season can provide a better water supply conditions together with precipitations [64]. For downstream areas, average annual precipitation is only 40 to 60 mm and average yearly potential evaporation rate reaches 2300 to 3700 mm [65], which lead to a significantly water stress condition and lower production values for GPP and NPP.

### Evaluation of the modelling results

Preliminary validation were performed to GPP rather than NPP due to lack of available field measurements of biomass in Heihe River Basin during our study period. In this study, we directly compared the modelled GPP with ground-based GPP derived from the twenty EC sites. We also adopted the MODIS GPP product (MOD17A2H) to assess our model performance. As MOD17A2H generates 8-day averaged GPP while our model produces 10-day averaged GPP, the MODIS GPP product was also compared to ground-based GPP this time.

**Fig 8:**
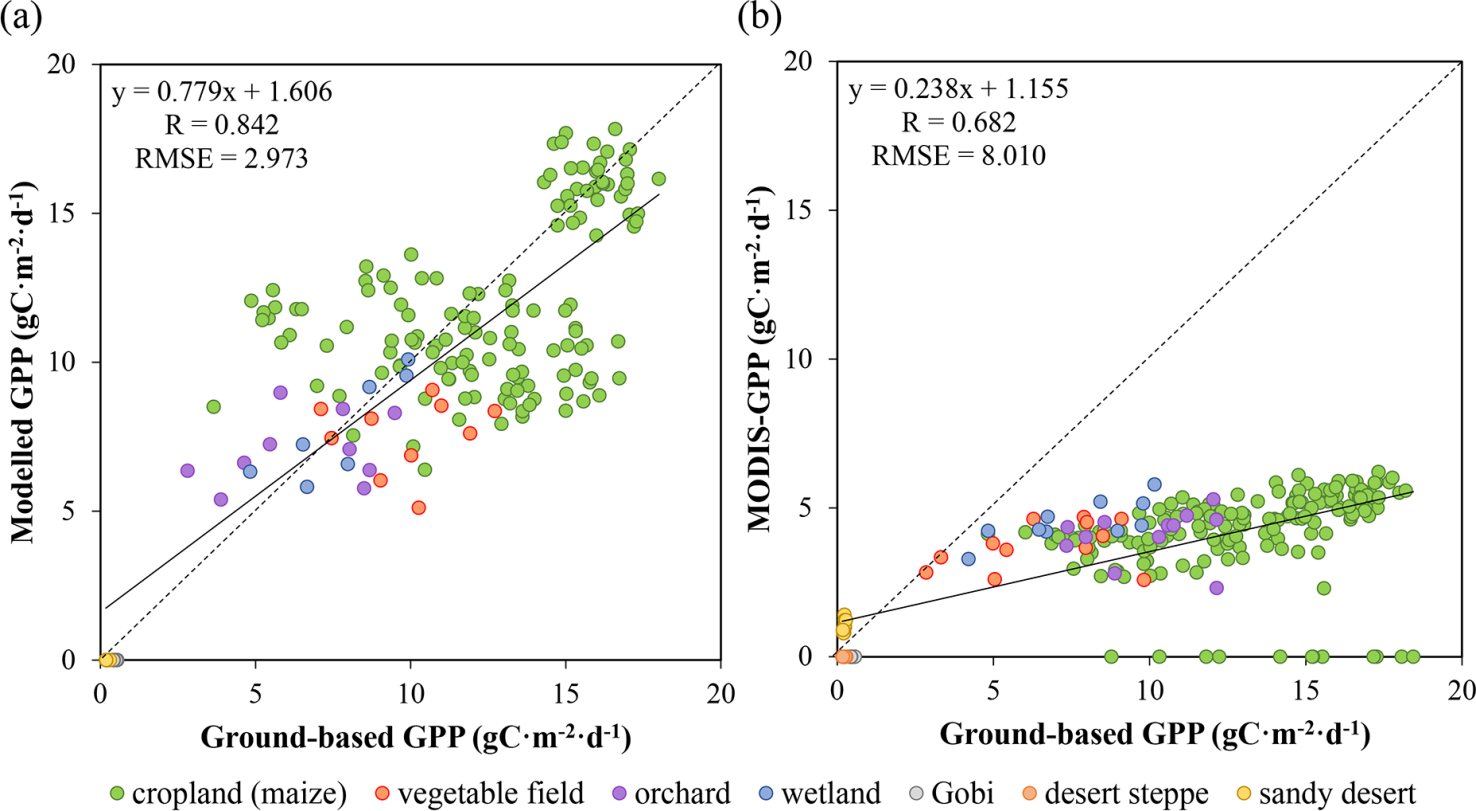
Model test against ground-based GPP. (a) Relationship between estimated GPP and ground-based GPP. (b) Relationship between MODIS GPP and ground-based GPP (b).

To decrease the co-registration errors between image and sampling sites, mean values in a 3 pixels × 3 pixels window around each sampling site were extracted as modelled results. Scatter plot between the modelled and ground-based GPP together with scatter plot between the MODIS GPP product and ground-based GPP are presented in Fig 8. As Fig 8 illustrates, an RMSE of 2.973 gC m^-2^ d^-1^ and an R of 0.842 can be yield between our modelled GPP and the ground-based measurements. Although a strong relationship exists between these two datasets, some individual pixels show relatively high scattering in the plot. The reason for the scattering can be attributed to several reasons. The most primary one is that we assumed LAI and FPAR hold constant within each month in our study, which may result in a mismatch between the monthly inputs and daily outputs. In addition, we directly compared the modelled GPP with ground measurements in this study, although ground surface at the EC sites and around them are relatively homogeneous for our study region, land cover categories will demonstrate significant variability within a 900 m × 900 m area. This kind of variation will definitely lead to some differences between modelled results and ground-based measurements. As for the MODIS GPP product, an RMSE of 8.010 gC m^-2^ d^-1^ and an R of 0.682 can be achieved. Scatter plot between the MODIS GPP product and ground-based GPP prove that the relationship between these two data sets deviate from the 1:1 line significantly: the former is generally lower than the latter, especially for croplands. The significant underestimation of MODIS GPP for croplands compared with our model can be attributed to the lower potential LUE used for C4 crops (mainly maize for our study area) in the MODIS GPP algorithm. As potential LUE for crops is assigned as 1.044 gC MJ^-1^ and shows no difference between C3 and C4 species in the MODIS GPP algorithm while C4 crops actually demonstrate a much higher potential LUE than C3 crops [54]. Additionally, the MODIS GPP algorithm utilizes the Collection 6 MODIS product for land cover type (MCD12Q1) in generating GPP. Since Collection 6 MCD12Q1 is designed at a 0.5 km grid scale, it can be difficult to obtain accurate land cover in areas with complex land surface conditions. For our study area, one cropland is misclassified as urban/built-up, which result in the incorrectly calculated GPP for the cropland.

To qualitatively evaluate the modelled NPP, we compiled a number of published studies carried out in our study region or similar biomes. The methods used to estimate NPP in these previous studies range from field investigation, simple regression analysis to LUE models, and cover a number of scales from monthly to yearly. Although these studies were carried out in different years, we assumed these results can qualitatively assess the MuSyQ-NPP results this time. The compilation of studies for Heihe River Basin have defined the range of NPP in the upstream region from 250 to 350 gC m^-2^ a^-1^ in 2002, 134 to 406 gC m^-2^ a^-1^ in 2010, and 100 to 500 gC m^-2^ a^-1^ in 2012 by referring to Lu et al. [66], Li et al. [67] and Zhang et al. [68] respectively. NPP can be greater than 720 gC m^-2^ a^-1^ in the midstream region and under 100 gC m^-2^ a^-1^ in the downstream region in 2002 [69]. For the whole region, NPP ranging from 0 to 801 gC m^-2^ a^-1^ in 2010, 3 to 987 gC m^-2^ a^-1^ in 2002 according to Li et al. [67] and Chen et al. [69], respectively. Additionally, we also took annual averaged NPP estimated for croplands and grasslands (891 and 237 gC m^-2^ a^-1^ in 2005, respectively) of China to evaluate our model [70]. Due to the temporal mismatch between these collected literature and the MuSyQ-NPP results, we assumed the accumulated NPP during growing season could account for more than 91.99% of the annual NPP for Heihe River Basin by referring to Zhang et al. [68]. Consequently, we considered annual NPP would range from 34 to 295 gC m^-2^ a^-1^, 482 to 921 gC m^-2^ a^-1^, and 25 to 149 gC m^-2^ a^-1^ in 2012 for upstream, midstream, and downstream region, respectively, when using our model. These values are highly consistent with values obtained in the previous literature.

## Discussion

With a focus on data that are openly accessible, we tested the presented MuSyQ-NPP algorithm for modelling daily GPP and NPP with 300 m spatial resolution for a 10-day period using multi-source and multi-scale data. MuSyQ-NPP algorithm adopted the theories of CASA model and MODIS GPP/NPP algorithm, we also considered the inherent limitations of these two approaches. The study would play an important role in the establishment of an appropriate model for calculating GPP and NPP at regional and global scale with relatively high spatial and temporal resolution.

One of the most important advantages of the presented MuSyQ-NPP algorithm is the improvements in model formulation and model inputs when compared with the widely used CASA model and MODIS GPP/NPP algorithm. CASA model adopts a biome-independent potential LUE of 0.389 gC MJ^-1^ and calculates NPP using the LUE theory, which is inadequate in characterizing the biological efficiency associated with biome types and the respiration property influenced by individual size. Compared with CASA model, MODIS GPP/NPP algorithm exploits a biome-specific potential LUE and calculates GPP and respiration items separately, which may address some aforementioned limitations of CASA. However, MODIS GPP/NPP algorithm deploys a potential LUE of 1.044 gC MJ^-1^ for crops without distinguishing C3 and C4 species while C4 crops generally demonstrate a much higher potential LUE than C3 crops. As for the MuSyQ-NPP algorithm, a potential LUE of 1.30 gC MJ^-1^ for C3 crops and a potential LUE value of 1.70 gC MJ^-1^ for C4 crops were used [54], which has improved the estimated GPP significantly when compared with the MODIS GPP product. It also should be noted that daily MODIS PSNnet does not include the calculation of growth respiration and maintenance respiration items associated with live wood when compared with NPP while the MuSyQ-NPP algorithm can provide the calculation for daily NPP, which would play an important role in the time series analysis of NPP.

**Fig 9:**
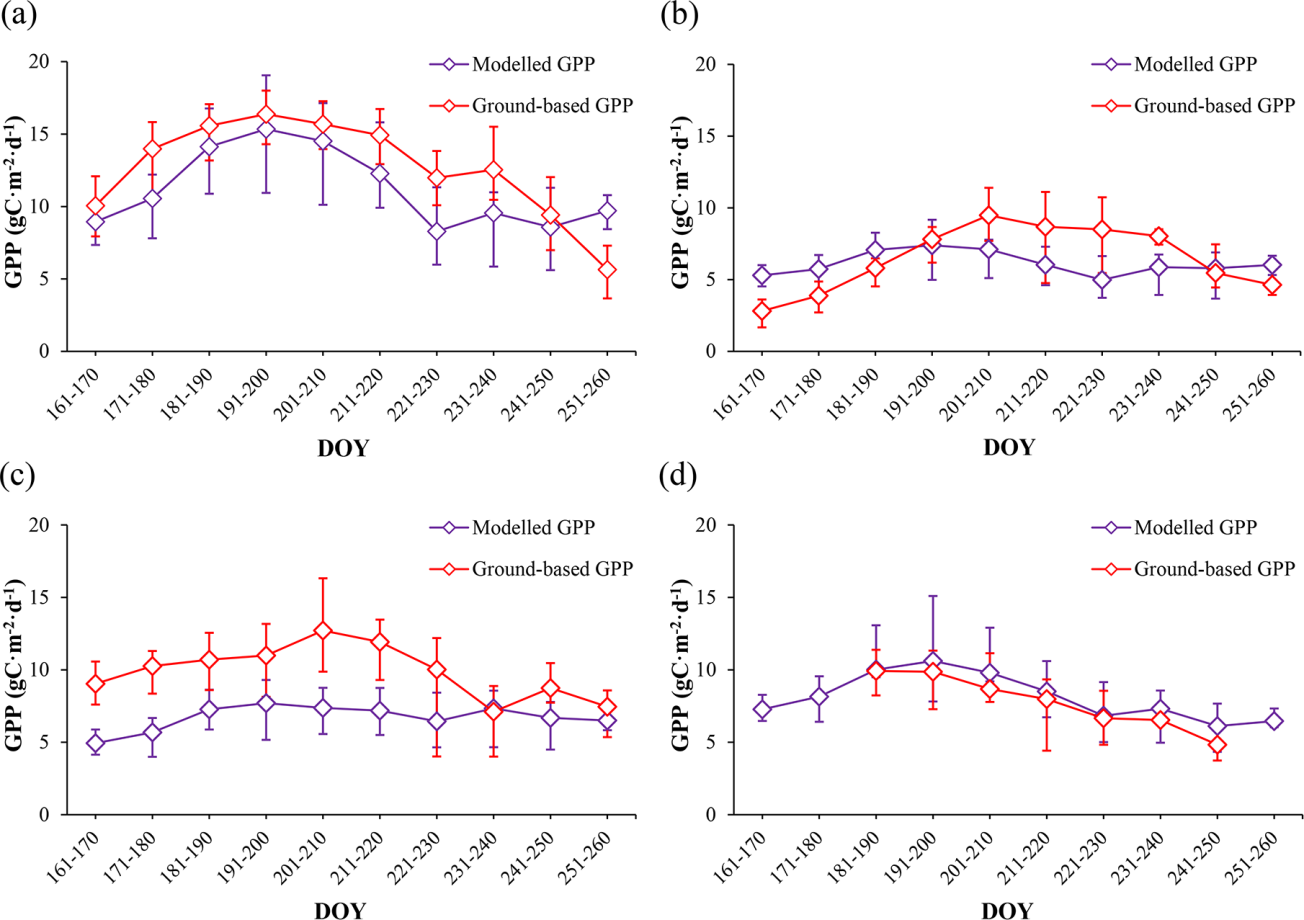
Temporal dynamic patterns of modelled GPP generated with simulated daily LAI and FPAR together with ground based ones during the growing season of 2012. (a) croplands. (b) orchard. (c) vegetable field. (d) wetland. For modelled and ground-based values, error bars represent mean and maximum/minimum for GPP in a 10-day period.

Another advantage of the MuSyQ-NPP algorithm is the exclusive use of multi-source and multi-scale data that are openly accessible. MODIS GPP/NPP algorithm adopts the Collection 6 MODIS products for LAI/FPAR and land cover type with spatial resolution of 0.5 km, it also deploys hourly meteorological data provided by the GMAO which are distributed at a resolution of 0.5° by 0.67°. Compared with the MODIS GPP/NPP algorithm, we occupied a series of remotely sensed and meteorological data with higher spatial resolutions in the MuSyQ-NPP algorithm. Among these data, remotely sensed data, such as LAI, FPAR, and land cover categories, are all designed at a 30 m spatial resolution, GLDAS based meteorological data are distributed at a spatial resolution of 0.25 degree. However, it should be noted that lower spatial resolution data including GLDAS-based meteorological data, DEM and forest biomass were all converted to higher spatial resolution using simple spatial interpolation algorithms. Both the quality of these input data and the interpolating methods will affect our estimated results, especially for the forest biomass data obtained from 1998 to 2003, which is quite different from our study period. The temporal mismatch between the forest biomass data used and our study period will introduce significant uncertainties. Additional studies should focus on the collection and adoption of data at more proper spatial and temporal resolution, such as the openly accessed NASA Shuttle Radar Topographic Mission (SRTM) 90m DEM data, which would significantly improve modelling results. The LAI and FPAR data used in this study have a temporal resolution of 1 month, which may be insufficient in characterizing their variations over monthly scale when used in generating daily GPP and NPP. To evaluate the impacts of monthly scaled inputs, we tested the MuSyQ-NPP algorithm based on simulated daily LAI and FPAR. Since daily LAI and FPAR were not available for our study area during the study period, in this study, daily LAI and FPAR were generated by performing linear interpolation algorithm to the monthly scaled data. We then calculated daily GPP based on the daily inputs (including daily LAI, FPAR and meteorological data). To compare with the previously generated 10-day averaged GPP, the simulated data were also plotted in a 10-day scale. As shown in Fig 9, the modelled GPP varied more continuous when used daily inputs. Additionally, the temporal dynamic patterns of modelled GPP were found to be better corresponded with the ground-based ones. We also noticed the modelled and observed GPP for orchard and vegetable field still demonstrated significant differences, which can be attributed to the spatial variability around these two EC sites. Fig 9 indicated that using LAI and FPAR at a more proper scale would help to address some model uncertainties caused by monthly inputs.

For our study, GPP was evaluated under an assumption that spatial heterogeneous characteristic near the EC sites could be neglected. Although ground surface at the EC sites and around them was relatively homogeneous for our study region, mismatches between modelled results and ground-based measurements should not be neglected. To tackle this drawback, footprint models together with scaling transfer functions can be deployed to improve our validation procedure in our future researches. Due to lack of field measurements, our validation was mainly performed to GPP this time, the modelled NPP were compared with results reported in other literatures in our study. It should be pointed that these two data sets may not obtained at the same time and locations. Additionally, all ground-based field data were collected in the midstream region of Heihe River Basin covered mainly by croplands in this study. As Heihe River Basin occupies different natural, ecological and climate characteristics in the upstream, midstream and downstream regions with various land cover categories, to characterize the spatial and temporal patterns of GPP and NPP in Heihe River Basin, further validation is still needed in assessing the accuracy of the MuSyQ-NPP algorithm.

## Conclusions

This study put forward the MuSyQ-NPP algorithm driven by remotely sensed data and meteorological data with spatial resolutions varying from 30 m to 0.25 degree and temporal resolutions ranging from 3 hours to 1 month to generate daily GPP and NPP for a 10-day period. The algorithm integrates remote sensing techniques and eco-physiological process theories. After validated against ground-based measurements and MODIS GPP in Heihe River Basin, we found the presented MuSyQ-NPP algorithm performed well in estimating GPP despite constant LAI and FPAR values within a month period were used. Additionally, the comparison with data derived from recent literature on NPP in Heihe River Basin or similar vegetation types also proofed the reliability of our model.

This study showed that both GPP and NPP vary significantly along the upstream, midstream, and downstream of Heihe River Basin from south to north during the growing period. The upstream regions occupy relatively low production, the midstream areas demonstrate the highest production, and the downstream regions show the lowest production. The mainly reasons for this kind of gradient distribution are attributed to temperature and water conditions of Heihe River Basin: the upstream undertakes a relatively low temperature which inhibits the growth of vegetation, the downstream suffers a significantly water stress condition, and the midstream occupies the best environmental and artificial management conditions for vegetation growth. During the growing period of 2012, GPP and NPP increase over time initially and decrease after reach their maximum around July. This is mainly driven by the seasonal variation of temperature, precipitation and solar influx conditions in the Heihe River Basin.

## References

[1] Liu J, Chen JM, Cihlar J, Chen W. Net primary productivity distribution in the BOREAS region from a process model using satellite and surface data. J Geophys Res: Atmos. 1999; 104: 27735–27754.

[2] Melillo JM, Mcguire AD, Kicklighter DW, Moore B, Vorosmarty CJ, Schloss AL. Global climate-change and terrestrial net primary production. Nature 1993; 363: 234–240.

[3] Propastin P, Kappas M. Modeling net ecosystem exchange for grassland in central Kazakhstan by combining remote sensing and field data. Remote Sens. 2009; 1: 159–183.

[4] Falge E, Baldocchi D, Tenhunen J, Aubinet M, Bakwin P, Berbigier P, et al Seasonality of ecosystem respiration and gross primary production as derived from FLUXNET measurements. Agr Forest Meteorol 2002; 113: 53–74.

[5] Wofsy SC, Goulden ML, Munger JW, Fan SM, Bakwin PS, Daube BC, et al Net exchange of CO_2_ in a midlatitude forest. Science 1993; 260: 1314–1317. 17755426 10.1126/science.260.5112.1314

[6] Prieto-Blanco A, North PRJ, Barnsley MJ, Fox N. Satellite-driven modelling of net primary productivity (NPP): Theoretical analysis. Remote Sens Environ. 2009; 113: 137–147.

[7] Lu DS. The potential and challenge of remote sensing-based biomass estimation. Int J Remote Sens. 2006; 27: 1297–1328.

[8] Myneni RB, Keeling CD, Tucker CJ, Asrar G, Nemani RR. Increased plant growth in the northern high latitudes from 1981 to 1991. Nature 1997; 386: 698–702.

[9] Turner DP, Ritts WD, Cohen WB, Gower ST, Zhao MS, Running SW, et al Scaling gross primary production (GPP) over boreal and deciduous forest landscapes in support of MODAS GPP product validation. Remote Sens Environ. 2003; 88: 256–270.

[10] Zhou LM, Tucker CJ, Kaufmann RK, Slayback D, Shabanov NV, Myneni RB. Variations in northern vegetation activity inferred from satellite data of vegetation index during 1981 to 1999. J Geophys Res: Atmos. 2001; 106: 20069–20083.

[11] Zhou Y, Zhu Q, Chen JM, Wang YQ, Liu J, Sun R, et al Observation and simulation of net primary productivity in Qilian Mountain, western China. J Environ Manage 2007; 85: 574–584. 17129660 10.1016/j.jenvman.2006.04.024

[12] Lieth H. Modeling the primary productivity of the world In: Lieth H, Whittaker RH, editors. Primary Productivity of the Biosphere. New York: Springer-Verlag Press; 1975 pp. 237–263.

[13] Uchijima Z, Seino H. Agroclimatic evaluation of net primary productivity of nature vegetation. (1). Chikugo model for evaluating primary productivity. J Agri Meteorol 1985; 40: 343–352.

[14] Potter CS, Randerson JT, Field CB, Matson PA, Vitousek PM, Mooney HA, et al Terrestrial ecosystem production: a process model-based on global satellite and surface data. Global Biogeochem Cy. 1993; 7: 811–841.

[15] Prince SD, Goward SN. Global primary production: A remote sensing approach. J Biogeogr. 1995; 22: 815–835.

[16] Xiao XM, Zhang QY, Braswell B, Urbanski S, Boles S, Wofsy S, et al Modeling gross primary production of temperate deciduous broadleaf forest using satellite images and climate data. Remote Sens Environ. 2004; 91: 256–270.

[17] Parton WJ, Scurlock JMO, Ojima DS, Gilmanov TG, Scholes RJ, Schimel DS, et al Observations and modeling of biomass and soil organic-matter dynamics for the grassland biome worldwide. Global Biogeochem Cy. 1993; 7: 785–809.

[18] McGuire AD, Melillo JM, Kicklighter DW, Joyce LA. Equilibrium responses of soil carbon to climate change: Empirical and process-based estimates. J Biogeogr. 1995; 22: 785–796.

[19] Running SW, Hunt ER. Generalization of a forest ecosystem process model for other biomes, BIOME-BGC, and an application for global-scale models In: Ehleringer JR, editor. Scaling Physiological Processes: Leaf to Globe. San Diego: Academic Press; 1993 pp. 141–158.

[20] Matsushita B, Xu M, Chen J, Kameyama S, Tamura M. Estimation of regional net primary productivity (NPP) using a process-based ecosystem model: How important is the accuracy of climate data? Ecol Model. 2004; 178: 371–388.

[21] Zhu WQ, Pan YZ, Zhang JS. Estimation of net primary productivity of Chinese terrestrial vegetation based on remote sensing. J Plant Ecol. 2007; 31: 413–424.

[22] Ruimy A, Saugier B, Dedieu G. Methodology for the estimation of terrestrial net primary production from remotely sensed data. J Geophys Res: Atmos. 1994; 99: 5263–5283.

[23] Liu J, Chen JM, Cihlar J, Park WM. A process-based boreal ecosystem productivity simulator using remote sensing inputs. Remote Sens Environ. 1997; 62: 158–175.

[24] Matsushita B, Tamura M. Integrating remotely sensed data with an ecosystem model to estimate net primary productivity in East Asia. Remote Sens Environ. 2002; 81: 58–66.

[25] Sun R, Zhu Q. Net primary productivity of terrestrial vegetation—a review on related rsearches. Chinese J Appl Ecol. 1999; 10: 757–760.

[26] Wang P, Sun R, Hu J, Zhu Q, Zhou Y, Li L, et al Measurements and simulation of forest leaf area index and net primary productivity in Northern China. J Environ Manage 2007; 85: 607–615. 17166651 10.1016/j.jenvman.2006.08.017

[27] Running SW, Zhao MS. User’s guide daily GPP and annual NPP (MOD17A2/A3) products NASA Earth Observing System MODIS land algorithm Version 3.0 for Collection 6. 2015.

[28] Running SW, Coughlan JC. A general-model of forest ecosystem processes for regional applications. 1. Hydrologic balance, canopy gas-exchange and primary production processes. Ecol Model 1988; 42: 125–154.

[29] Chen JM, Liu J, Cihlar J, Goulden ML. Daily canopy photosynthesis model through temporal and spatial scaling for remote sensing applications. Ecol Model 1999; 124: 99–119.

[30] Feng X, Liu G, Chen JM, Chen M, Liu J, Ju WM, et al Net primary productivity of China's terrestrial ecosystems from a process model driven by remote sensing. J Environ Manage. 2007; 85: 563–573. 17234327 10.1016/j.jenvman.2006.09.021

[31] Pachavo G, Murwira A. Remote sensing net primary productivity (NPP) estimation with the aid of GIS modelled shortwave radiation (SWR) in a Southern African Savanna. Int J Appl Earth Obs. 2014; 30: 217–226.

[32] Piao SL, Fang JY, Guo QH. Application of CASA model to the estimation of Chinese terrestrial net primary productivity. J Plant Ecol. 2001; 25: 603–608.

[33] Turner DP, Urbanski S, Bremer D, Wofsy SC, Meyers T, Gower ST, et al A cross-biome comparison of daily light use efficiency for gross primary production. Global Change Biol. 2003; 9: 383–395.

[34] Field CB, Randerson JT, Malmstrom CM. Global net primary production—combining ecology and remote-sensing. Remote Sens Environ. 1995; 51: 74–88.

[35] Ma MG, Frank V. Interannual variability of vegetation cover in the Chinese Heihe River Basin and its relation to meteorological parameters. Int J Remote Sens. 2006; 27: 3473–3486.

[36] Wang XF, Ma MG, Huang GH, Veroustraete F, Zhang ZH, Song Y, et al Vegetation primary production estimation at maize and alpine meadow over the Heihe River Basin, China. Int J Appl Earth. Obs. 2012; 17: 94–101.

[37] Liu B, Zhao WZ, Chang XX, Li SB, Zhang ZH, Du MW. Water requirements and stability of oasis ecosystem in arid region, China. Environ Earth Sci. 2010; 59: 1235–1244.

[38] Li X, Lu L, Cheng GD, Xiao HL. Quantifying landscape structure of the Heihe River Basin, north-west China using FRAGSTATS. J Arid Environ. 2001; 48: 521–535.

[39] Pan X.D, Li X, Yang K, He J, Zhang YL, Han XJ. Comparison of downscaled precipitation data over a mountainous watershed: A case study in the Heihe River Basin. J Hydrometeorol. 2014; 15: 1560–1574.

[40] Fan WJ, Liu Y, Xu X.R, Chen GX, Zhang BT. A new FAPAR analytical model based on the law of energy conservation: A case study in China. IEEE J Stars. 2014; 7: 3945–3955.

[41] Liao YR, Fan WJ, Xu XR. Algorithm of leaf area index product for HJ-CCD over Heihe River Basin. Geoscience and Remote Sensing Symposium (IGARSS) 2013 IEEE International. IEEE 2013; 169–172.

[42] Zhong B, Ma P, Nie AH, Yang AX, Yao YJ, Lu WB, et al Land cover mapping using time series HJ-1/CCD data. Sci China Earth Sci. 2014; 57: 1790–1799.

[43] Rodell M, Houser P.R, Jambor U, Gottschalck J, t K, Meng CJ, Arsenault K, Cosgrove B, Radakovich J, Bosilovich M. et al The global land data assimilation system. B Am Meteorol Soc. 2004; 85: 381–394.

[44] Weng DM, Sun ZA. A preliminary study of the lapse rate of surface air temperature over mountainous regions of China. Geophys Res. 1984; 3: 24–34.

[45] Frouin R, Pinker RT. Estimating photosynthetically active radiation (Par) at the earths surface from satellite-observations. Remote Sens Environ. 1995; 51: 98–107.

[46] Zhu HZ. Classification of vegetation and variations in carbon density in forests of China based on ecological process parameters and remote sensing. D.Sc. Thesis, Graduate School of Chinese Academy of Sciences. 2006. Available: http://d.wanfangdata.com.cn/Thesis/Y1627345

[47] Li X, Cheng GD, Liu SM, Xiao Q, Ma MG, Jin R, et al Heihe Watershed Allied Telemetry Experimental Research (HiWATER): Scientific objectives and experimental design. B Am Meteorol Soc. 2013; 94: 1145–1160.

[48] Xu ZW, Liu SM, Li X, Shi SJ, Wang JM, Zhu ZL, et al Intercomparison of surface energy flux measurement systems used during the HiWATER-MUSOEXE. J Geophys Res: Atmos. 2013; 118: 13140–13157.

[49] Liu SM, Xu ZW, Wang WZ, Jia ZZ, Zhu MJ, Bai J, et al A comparison of eddy-covariance and large aperture scintillometer measurements with respect to the energy balance closure problem. Hydrol Earth Syst Sci. 2011; 15: 1291–1306.

[50] Coops NC, Black TA, Jassal RPS, Trofymow JAT, Morgenstern K. Comparison of MODIS, eddy covariance determined and physiologically modelled gross primary production (GPP) in a Douglas-fir forest stand. Remote Sens Environ. 2007; 107: 385–401.

[51] Wang HM, Saigusa N, Yamamoto S, Kondo H, Hirano T, Toriyama A, et al Net ecosystem CO_2_ exchange over a larch forest in Hokkaido, Japan. Atmos Environ. 2004; 38: 7021–7032.

[52] Zhang L, Sun R, Liu SM, Xu ZW, Qiao C, Jiang GQ. Diurnal and seasonal variations in carbon dioxide exchange in ecosystems in the Zhangye oasis area Northwest China. PLOS One. 2015; 10(3): e0120660 doi: 10.1371/journal.pone.0120660 25803840 10.1371/journal.pone.0120660 PMC4372606

[53] Monteith JL. Solar radiation and productivity in tropical ecosystems. J Appl Ecol. 1972; 9: 747–766.

[54] Sinclair TR, Horie T. Leaf nitrogen, photosynthesis, and crop radiation use efficiency: A review. Crop Sci. 1989; 29: 90–98.

[55] Monteith JL. Evaporation and environment. Symp Soc Exp Biol. 1965; 19: 205–234. 5321565

[56] Zhang K, Kimball JS, Nemani RR, Running SW. A continuous satellite-derived global record of land surface evapotranspiration from 1983 to 2006. Water Resour Res. 2010; 46.

[57] Qiao C, Sun R, Xu ZW, Zhang L, Liu LY, Hao LY, Jiang GQ. A study of shelterbelt transpiration and cropland evapotranspiration in an irrigated area in the middle reaches of the Heihe River in northwestern China. IEEE Geosci Remote S. 2015; 12: 369–373.

[58] Zhang K, Kimball JS, Mu QZ, Jones LA, Goetz SJ, Running SW. Satellite based analysis of northern ET trends and associated changes in the regional water balance from 1983 to 2005. J Hydrol. 2009; 379: 92–110.

[59] Los SO, Collatz GJ, Sellers PJ, Malmstrom CM, Pollack NH, DeFries RS, et al A global 9-yr biophysical land surface dataset from NOAA AVHRR data. J Hydrometeorol. 2000; 1: 183–199.

[60] Mu QZ, Zhao MS, Running SW. Improvements to a MODIS global terrestrial evapotranspiration algorithm. Remote Sens. Environ. 2011; 115: 1781–1800.

[61] Zhang YQ, Chiew FHS, Zhang L, Leuning R, Cleugh HA. Estimating catchment evaporation and runoff using MODIS leaf area index and the penman-monteith equation. Water Resour Res. 2008; 44.

[62] Priestley CHB, Taylor RJ. On the assessment of surface heat flux and evaporation using large-scale parameters. Monthly weather review 1972; 100: 81–92.

[63] Xiao Q, Wen JG. HiWATER: visible and near-infrared hyperspectral radiometer (29th, June, 2012) Institute of Remote Sensing and Digital Earth, Chinese Academy of Sciences 2012.

[64] Fan L, Xiao Q, Wen JG, Liu Q, Tang Y, You DQ, et al Evaluation of the airborne CASI/TASI Ts-VI space method for estimating near-surface soil moisture. Remote Sens. 2015; 7: 3114–3137.

[65] Li XQ, Gan YQ, Zhou AG, Liu YD, Wang D. Hydrological controls on the sources of dissolved sulfate in the Heihe River a large inland river in the arid northwestern China, inferred from S and O isotopes. Appl Geochem. 2013; 35: 99–109.

[66] Lu L, Li X, Veroustraete F. Estimation of net primary productivity of Heihe River Basin using remote sensing. J Desert Res. 2005; 25: 823–830.

[67] Li XP. Remotely-sensed estimation of NPP and its spatial-temporal characteristics in the Heihe River Basin. M.Sc. Thesis, Shaanxi Normal University. 2013. Available: http://cdmd.cnki.com.cn/Article/CDMD-10718-1014108318.htm

[68] Zhang YS, Jia WX, Zhao YF, Liu YR, Zhao Z, Chen JH. Spatial-temporal variations of net primary productivity of Qilian mountains vegetation based on CASA model. Acta Bot Boreal Occident Sin. 2014; 34: 2085–2091.

[69] Chen ZH, Ma QY, Wang J, Qi Y, Li J, Huang CL, et al Estimation of Heihe basin net primary productivity using the CASA model. J Natural Res. 2008; 23: 263–273.

[70] Zhang HL. The feature analysis on China’s terrestrial NPP spatial-temporal change in recent five years. M.Sc. Thesis, Nanjing Normal University. 2006. Available: http://cdmd.cnki.com.cn/Article/CDMD-10319-2006154235.htm

